# Colorimetric detection of Cu^2+^ based on the formation of peptide–copper complexes on silver nanoparticle surfaces

**DOI:** 10.3762/bjnano.9.134

**Published:** 2018-05-15

**Authors:** Gajanan Sampatrao Ghodake, Surendra Krishna Shinde, Rijuta Ganesh Saratale, Avinash Ashok Kadam, Ganesh Dattatraya Saratale, Asad Syed, Fuad Ameen, Dae-Young Kim

**Affiliations:** 1 Department of Biological and Environmental Science, College of Life Science and Biotechnology, Dongguk University-Seoul, Ilsandong-gu, 10326, Goyang-si, Gyeonggi-do, Republic of Korea; 2 Research Institute of Biotechnology & Medical Converged Science, Dongguk University-Seoul, 32, Dongguk-ro, Ilsandong-gu, Goyang-si, Gyonggido, 10326, Republic of Korea; 3 Department of Food Science and Biotechnology, Dongguk University-Seoul, Ilsandong-gu, Goyang-si, Gyeonggido, 10326, Republic of Korea; 4 Department of Botany and Microbiology, College of Science, King Saud University, P.O. 2455, Riyadh 11451, Saudi Arabia

**Keywords:** absorbance, dispersion, drinking water, rapid detection, UV spectroscopy

## Abstract

We developed a colorimetric method for the rapid detection of copper ions (Cu^2+^) in aqueous solution. The detection of Cu^2+^ is based on coordination reactions of Cu^2+^ with casein peptide-functionalized silver nanoparticles (AgNPs), leading to a distinct color change of the solution from yellow to red. The developed method has a good detection limit of about 0.16 µM Cu^2+^ using 0.05 mL of AgNPs stock solution and a linearity in the range of 0.08–1.44 µM Cu^2+^ with a correlation coefficient of R^2^ = 0.973. The developed method is a useful tool for the detection of Cu^2+^ ions. Furthermore, it can be used for monitoring Cu^2+^ in water at concentrations below the safe limit for drinking water set by the World Health Organization.

## Introduction

Among heavy metals, copper is particularly interesting because it acts both as a micronutrient essential to life, but also as an environmental contaminant, due to its toxicity to most organisms when present above certain concentrations [1]. Copper is a widely distributed heavy metal in water bodies, as a result of direct dumping of industrial and mining waste and electronic waste [2–3]. Cu^2+^ has been used excessively in the form of copper sulfate and copper hydroxide as a fungicide, algaecide, and soil amendment, which also contributes to the increased Cu^2+^ concentrations [4]. Thus, a rapid and convenient approach for on-site visual analysis of Cu^2+^ is considered an important research topic in analytical chemistry [5]. However, procedures that rely on expensive and specialized equipment (e.g., atomic emission spectrometer, atomic absorption spectrometer, inductively coupled plasma mass spectrometer) are only of limited use for on-site applications [6]. Thus, the development of a convenient but highly sensitive and selective sensing method for Cu^2+^ with improved practicality is urgently needed.

Although colorimetric probes based on the aggregation of nanoparticles (NPs) induced by target-metal ions are advantageous, these probes may have difficulties in detecting Cu^2+^ in complex matrices [7]. Quantum-dot-based fluorescent probes showed improved sensitivity and selectivity for Cu^2+^ compared to traditional organic probes [8–10], but the preparation of such functionalized quantum dots is a time-consuming process. Similarly, colorimetric methods based on Cu^2+^-dependent click chemistry are well-known for high selectivity and tolerance to interference caused by other metal ions. Nonetheless, these strategies have limited practical applications due to a relatively high detection limit (3.0–20 μM) [11], which is much higher than that of fluorescence methods (0.8 μM) [5]. Recently, colorimetric methods based on gold [12], and platinum NPs have been widely reported for on-site use and analysis of multiple samples [13–14]. However, the use of gold and/or platinum limits the affordability of sensing probe. As exemplified in this work, cost-effective silver nanoparticles (AgNPs) having specifically modified ligands for detecting lower concentrations of Cu^2+^ offer a more portable and practical approach.

In a typical experiment, casein peptide-modified AgNPs were prepared by reduction of AgNO_3_ and the functionality of the AgNP–peptide conjugates to coordinate Cu^2+^ was improved by removing unbound casein peptides. Highly dispersed and stable 20 nm diameter AgNPs had an extinction peak at about 410 nm with a characteristic yellow appearance. In the presence of Cu^2+^, the coordination product was formed, followed by assembly of the AgNPs into aggregates, which exhibited extinction at 520 nm. In a proof-of-concept detection experiment, the estimated Cu^2+^ concentration range was 0.08–1.44 µM, depending on the amount of Cu^2+^-binding casein peptide ligands. The solution of aggregates was incubated for 20 min to allow for the coordination to occur.

## Results and Discussion

### Synthesis and characterization of casein peptide-capped AgNPs

The surface plasmon resonance (SPR) of spherical AgNPs immediately caused an absorbance peak in the UV–vis spectra at approximately 410–420 nm. The wavelength of maximum absorbance (λ_max_) can be used to determine the approximate concentration and the size range of the stable AgNPs. Herein, synthesis of AgNPs was performed using just 0.06% (w/v) casein peptides in the solution, to successfully convert 1 mM AgNO_3_ to crystalline AgNPs having a λ_max_ of 400 nm. No additional reducing/stabilizing agents were added to the solution. Thus, most of the green chemistry principles were followed during the preparation of casein peptide-functionalized AgNPs [15–16].

**Figure 1:**
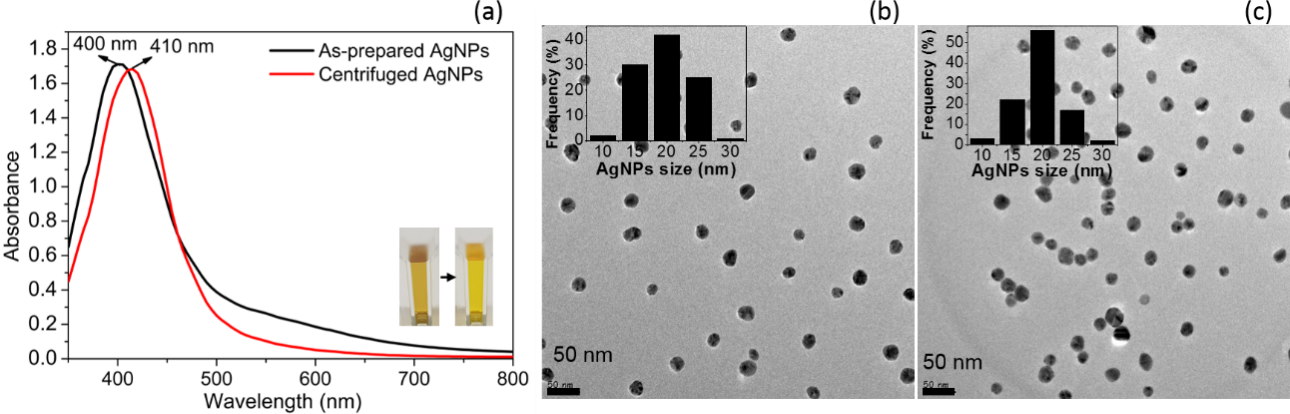
(a) UV–vis spectrum and color of silver nanoparticles before and after centrifugation. A representative HR-TEM image of silver nanoparticles (b) before and (c) after centrifugation.

The casein peptide-AgNPs were monodisperse in nature with a narrow size distribution, as expected from the narrow absorbance peak centered at 410 nm. The UV–vis spectroscopic data were collected before and after centrifugation (10000 rpm for 12 min) to observe how the SPR peak width and shift changed with the removal of unbound/unreacted casein peptides (Figure 1a). The minor red-shift (from 400 to 410 nm) and changes in the SPR peak width are indicative of the stable and dispersed nature of the AgNPs. Both peaks remained narrow, indicating a more homogeneous size distribution. Moreover, the absorption measurements revealed the stability of the casein peptide-facilitated functionality of the AgNPs (Figure 1a). These results are a qualitative measure of the size and size distributions of the AgNPs in distilled water after centrifugation.

Size, morphology, crystalline structure, and surface capping of the prepared AgNPs were characterized by high-resolution transmission electron microscopy (HR-TEM), XPS, XRD, and Fourier-transform infrared (FTIR) spectroscopy. The shape of the SPR peak determined the qualitative characterization of the AgNPs, and the size range of the particles in solution concurred with the HR-TEM images. As can be seen in the HR-TEM image, the freshly produced AgNPs had a highly uniform appearance with an average size of 20 ± 2 nm (Figure 1b). A representative HR-TEM image of the AgNPs after separation of excess casein peptides is also shown (Figure 1c). After centrifugation and re-dispersion of the AgNPs, some aggregates appeared, whereas, the average size of the individual AgNPs remained unchanged (Figure 1c). Accordingly, we concluded that under centrifugation conditions, bilayer structures of casein peptides remain intact and stable, thereby inhibiting AgNP aggregation and enabling increased sensitivity toward Cu^2+^ ions.

**Figure 2:**
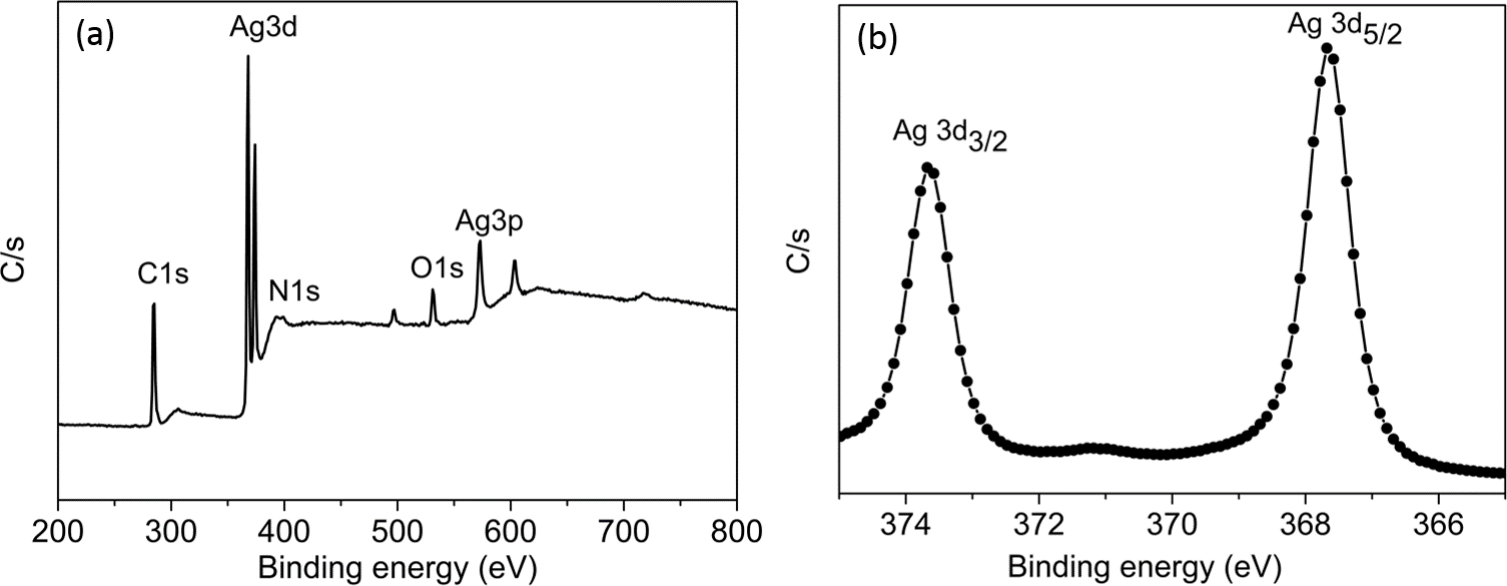
(a) XPS survey and (b) high-resolution Ag 3d spectra of casein peptide-capped silver nanoparticles.

The C 1s, O 1s, N 1s, and Ag 3d peaks originating from the AgNPs surface are shown in the XPS survey spectrum (Figure 2a). The presence of O1s and N1s peaks after centrifugation indicates that the capping with casein peptides through electrostatic interaction was stable and appropriate to develop analytical applications. It can be concluded that amino acids bind to the nanoparticle surfaces through NH_2_–OH hydrogen bonds and electrostatic interactions [17]. In the N 1s spectrum two peaks were identified. The peak at 398.2 eV was allocated to deprotonated nitrogen bindings (N–C=O and NH_2_) while the higher binding energy (BE) peak at 402.0 eV corresponds to protonated amino groups [18]. The Ag 3d XPS spectrum (Figure 2b) shows two prominent spin–orbit pairs at 367.6 (Ag 3d_5/2_) and 373.5 (Ag 3d_3/2_) eV, separated by 5.9 eV, and a broadening at high BE values also occurred. The first spin–orbit pair of small intensity at higher BE values is due to the oxidized Ag surface atoms bonding with the peptide molecule. The main signal, the Ag 3d_5/2_ component, centered at 367.6 eV BE, corresponds to metallic Ag atoms in the bulk of AgNPs [19].

**Figure 3:**
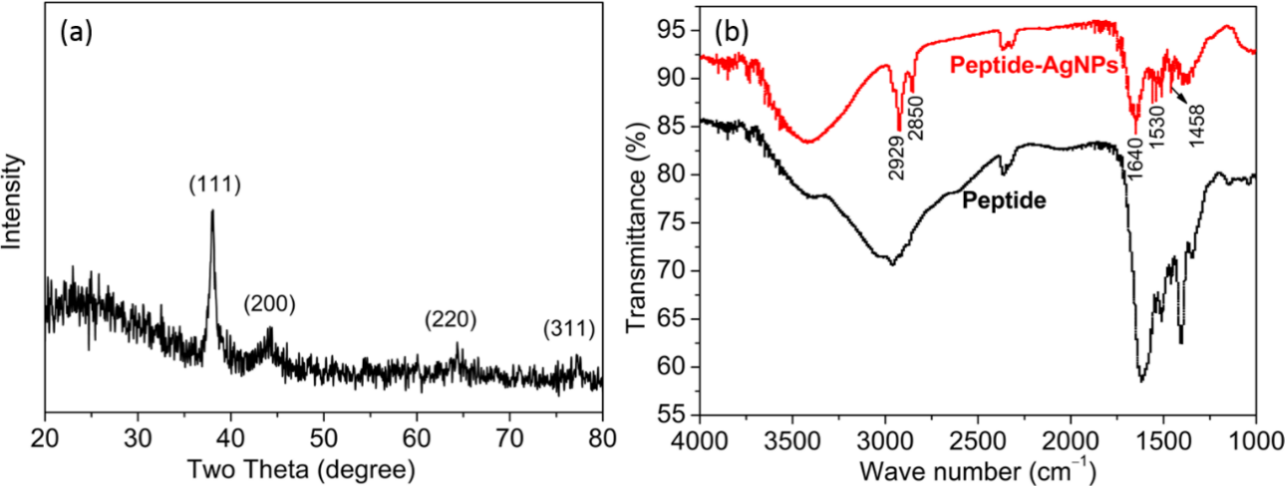
(a) XRD pattern and (b) FTIR spectra of casein peptide-capped silver nanoparticles.

XRD was performed to reveal the formation of AgNPs and to identify their crystalline structure (Figure 3a). The diffraction peaks at scattering angles, 2θ, of 38.25°, 44.42°, 64.51° and 77.55° were assigned to the crystallographic planes (111), (200), (220) and (311), respectively.

FTIR was performed to examine the capping of casein peptides on the AgNP surfaces. The higher-frequency absorption band at 1640 cm^−1^, corresponding to the amide-I band is identified for centrifuged AgNPs (Figure 3b) while a lower-frequency amide-II band was shifted from 1620 to 1530 cm^−1^ after capping of casein peptides to AgNPs, largely due to *trans*-NH bending of the carbonyl oxygen. In this study, casein peptides were successfully used as reducing and stabilizing agents in the formation of extremely stable AgNPs. Amide groups and side chains of the peptides were involved as both reducing and capping agents. Functional groups of casein peptides can result in passivation of the AgNP surface [20] and stabilize NPs via coordination of N atoms with Ag atoms at the surface of AgNPs [21–22]. Contributions of the side-chain functional groups of the casein peptides, such as C–H stretching modes and –CH_2_– bending modes were identified at 2929 and 2850 cm^−1^ and at 1458 cm^−1^, respectively, in agreement with [23].

### Selectivity

At first, the selectivity of the functionalized AgNPs was examined with various ions, including Cu^2+^, Mn^2+^, Mo^3+^, Na^+^, Cr^3+^, Hg^2+^, Ni^2+^, Ca^2+^, K^+^, Cs^+^, Li^+^, As^+^, PO_4_^3−^, NH_4_, and NO_3_^−^, at 100 ppb. The color change of the AgNPs solutions was observed in the presence of various metal ions. The Cu^2+^ ions caused a change from yellow to red. However, no color change was noticed for the other ions and salts, and the original color and SPR band of the AgNPs were retained (data not shown). It was shown that casein peptide-capped AgNPs are suitable to detect Cu^2+^ ions with excellent selectivity, whereas the other evaluated metal ions exhibit insignificant coordination under identical conditions.

### Effect of acidic and alkaline conditions and ionic strength

**Figure 4:**
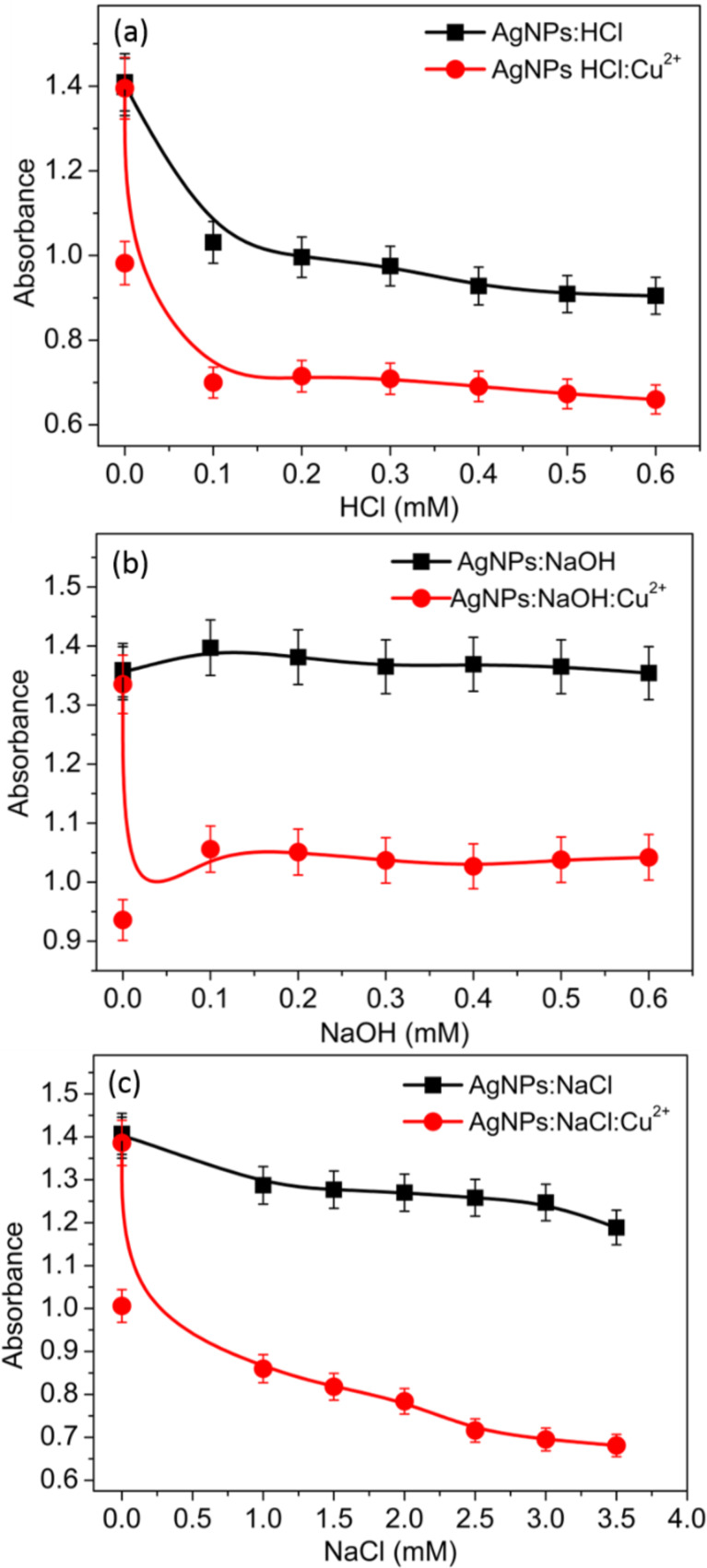
Absorbance intensity (at 410 nm) of the proposed probe in the presence and absence of Cu^2+^ at different (a) HCl concentrations, (b) NaOH concentrations and (c) ionic strengths.

In general, the pH value of the NP solution plays a vital role in the colorimetric detection. Consequently, the Cu^2+^-sensing ability of the functionalized AgNPs under increasingly acidic or alkaline conditions was investigated. The casein peptides led to AgNP aggregation under acidic conditions (0.1–0.6 mM HCl). However, alkaline conditions (0.1–0.6 mM NaOH) provided additional stability to the AgNP dispersions. The absorbance signal at 410 nm linearly decreased with increasing concentration of diluted HCl from 0.1 to 0.6 mM and then further declined in the presence of Cu^2+^. The extreme decrease in absorbance of AgNPs triggered by Cu^2+^ addition, even in the presence of 0.1 mM HCl, indicates that the functionalized AgNPs are very sensitive to acidic conditions (Figure 4a). The result showed that the AgNP response toward Cu^2+^ could be increased with an increase in acidity strength. Free –NH_2_/–COOH groups are more suitable to bind to divalent metal ions, such as Cu^2+^, as illustrated in [24].

In contrast, the absorbance intensity at 410 nm did not change markedly, before and after addition of Cu^2+^, with increasing concentration of NaOH (0.1 to 0.6 mM), demonstrating the stability of the analytical platform under alkaline pH conditions (Figure 4b). Under highly alkaline conditions, stable dispersions of AgNPs could be formed through interactions with OH^−^ ions [25], which inhibit the formation of AgNPs aggregates in the presence of Cu^2+^. Instead, in the present study, stable sensitivity occurred under alkaline pH conditions, possibly through the formation of various coordination complexes.

The ionic strength of the NP solution is also an important parameter during the detection of target metal ions. The effect of ionic strength on the absorbance of AgNPs is presented in Figure 4c. The absorbance intensity at 410 nm changed linearly before and after Cu^2+^ addition with increasing concentration of NaCl (1.0 to 3.5 mM), revealing that the sensitivity of the analytical platform can be increased with the increase of ionic strength. Based on these results, further tests on Cu^2+^ were performed using distilled water, even though this method is versatile and adaptable at different pH values and ionic strengths.

### Cu^2+^ quantification

It is highly desirable to establish a system based on visual and spectral detection of Cu^2+^ in water at concentrations below the safe limit for drinking water of 20 and 30 µM set by the US Environmental Protection Agency and the World Health Organization [5,26], and below the normal blood concentration of Cu^2+^, which is in the range of 24–135 µg/dL [27]. The interaction of Cu^2+^ with the peptide ligands on the AgNP surfaces attracts neighboring AgNPs, which is observed as a color change from yellow to red. The change in absorbance was monitored by UV–vis spectroscopy at various Cu^2+^ concentrations (0.08 to1.44 µM).

**Figure 5:**
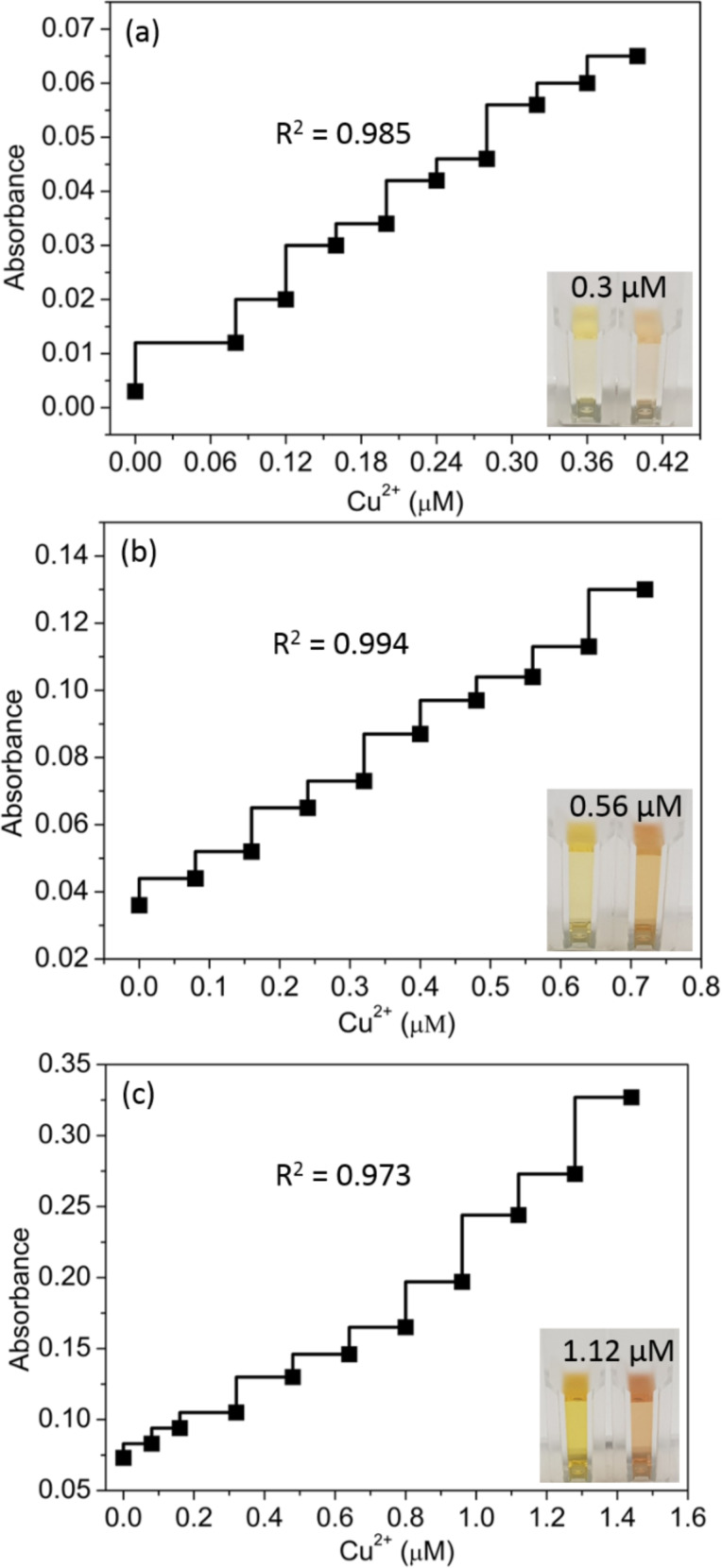
Absorbance intensity (at 520 nm) with increasing amounts of Cu^2+^ using (a) 0.05, (b) 0.1, and (c) 0.2 mL AgNPs solution. (The visual detection limits are given in the insets).

The absorbance intensity at 520 nm increased with an increase of the Cu^2+^ concentration, and the sensitivity was purely dependent upon the volume of added AgNPs stock solution as presented in Figure 5. Thus, the quantitative features, including the calibration curve, correlation coefficients (R^2^), and limit of detection (LOD) were studied using coordination peptides number approach. In all three instances, a linear correlation existed between the absorbance at 520 nm and the Cu^2+^ concentration over the ranges of 0.08–0.4, 0.08–0.72, and 0.08–1.44 µM Cu^2+^ (for 0.05, 0.1, and 0.2 mL of added AgNPs stock solution) with R^2^ = 0.985, 0.994, and 0.973, respectively (Figure 5). The spectral LOD toward Cu^2+^ was observed at about 0.16, 0.24, and 0.32 µM, respectively, for 0.05, 0.1, and 0.2 mL of added AgNPs solution with a broad detection range of 0.08–1.44 µM. The color of the solution changed from yellow to red by the addition of Cu^2+^ ions. The visual LOD is a desired characteristic of sensing probes. In this study, the visual LOD values of 0.32, 0.56, and 1.12 µM, respectively, for 0.05, 0.1, and 0.2 mL of stock AgNPs could be achieved, as shown in the insets of Figure 5. The increase in absorbance intensity at 520 nm was successfully used to quantify Cu^2+^, with a low intensity associated with the yellow dispersed AgNPs and a high intensity associated with the red colored aggregates of AgNPs. Moreover, these changes can be quantified with the naked eye. Involvement of the deprotonated amide groups in the coordination of Cu^2+^ was recently reported [28]. The LOD of the casein peptide-AgNPs is much lower than that of comparable methods (Table 1). A distinct color change was detectable by the naked eye at the smallest Cu^2+^ concentration of 0.4 μM. This concentration is significantly lower than that of 20 μM reported by Xu et al. [11], and 5.0 μM by Lu and Liu [26], using DNA-modified NPs. Recently, colorimetric methods based on gold NPs have been widely reported for Cu^2+^ detection (Table 1). However, as mentioned above, such procedures are costly. The detection platform established in the current report was sufficiently sensitive and cost-effective to detect Cu^2+^ in drinking water below the limit (20 μM) directed by the WHO and the United States Environmental Protection Agency [26].

**Table 1:** Detection limit of Cu^2+^ reported using various colorimetric methods and surface chemistries.

surface chemistry	nanomaterial	detection range (μM)	detection limit (μM)	reference

DNA	gold nanoparticles	20–100	20	[11]
DNA	gold nanoparticles	1–100	10	[26]
triazole	click chemistry	0.60–13	10	[35]
polythiophene	click chemistry	0.5–10	3.0	[5]
azide	gold nanoparticles	1.8–200	1.8	[36]
dopamine	gold nanoparticles	1–10	1.4	[24]
catalytic leaching	gold nanoparticles	0.03–3.0	0.7	[37]
copper catalysis	silver nanoparticles	0.25–2.0	0.75	[38]
casein peptide	silver nanoparticles	0.08–1.44	0.16	described here

### Stability of coordination complexes

**Figure 6:**
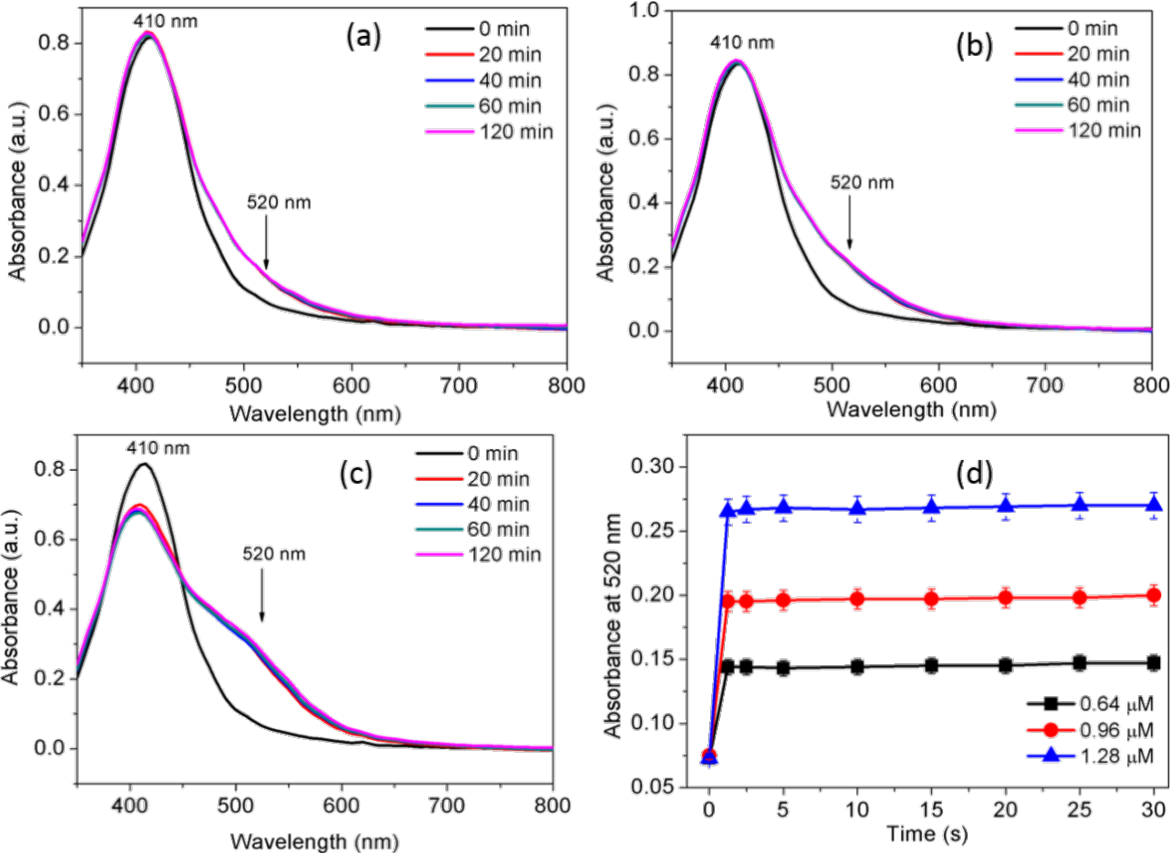
Time course of the spectral response of silver nanoparticles in the presence of (a) 0.64, (b) 0.96, and (c) 1.28 µM Cu^2+^. (d) Time course of the absorbance intensity of silver nanoparticles recorded in the presence of the three different Cu^2+^ concentrations.

Most of the colorimetric and visual methods are based on the change in color, and the spectral response received immediately after exposure to the target metal ions. The current report also studied the real-time UV–vis absorption spectroscopy, with the intent of investigating the stability of the established sensing platform. Stable spectral shifts indicate that the AgNPs–casein peptide probe is viable for rapid and sensitive detection of Cu^2+^. The SPR peak shift from 410 nm to longer wavelengths (520 nm) demonstrated the formation of the stable Cu^2+^ coordination complex at both low and high Cu^2+^ concentrations. Figure 6a–c presents the spectral response time of the AgNPs-casein peptides at 0.64, 0.96, and 1.28 µM Cu^2+^ within a time frame of 20 to 120 min. Initially, the absorbance intensity increased to a maximum and then remained stable for an extended period (120 min). The stability of sensing probe is noticed as no gradual decrease or increase in the absorbance was detected at all tested concentrations of Cu^2+^. In contrast, in an earlier report, the realization of fractal growth of the AuNP aggregates occurred for tryptophan–Mg^2+^ coordination complexes [29].

As can be seen in Figure 6d, the absorbance intensity at 520 nm increased rapidly within 1 s after exposure to different dosages of Cu^2+^ (0.64, 0.96, and 1.28 µM) and subsequently reached a stable value. In the presence of Cu^2+^, aggregation and a red appearance occurred almost within the fraction of a second. Therefore, the absorbance probe ensured the rapid detection of Cu^2+^, similar to fluorescent probes developed for the analysis of Fe^3+^ [30].

### Cu^2+^-spiked water samples analysis

**Figure 7:**
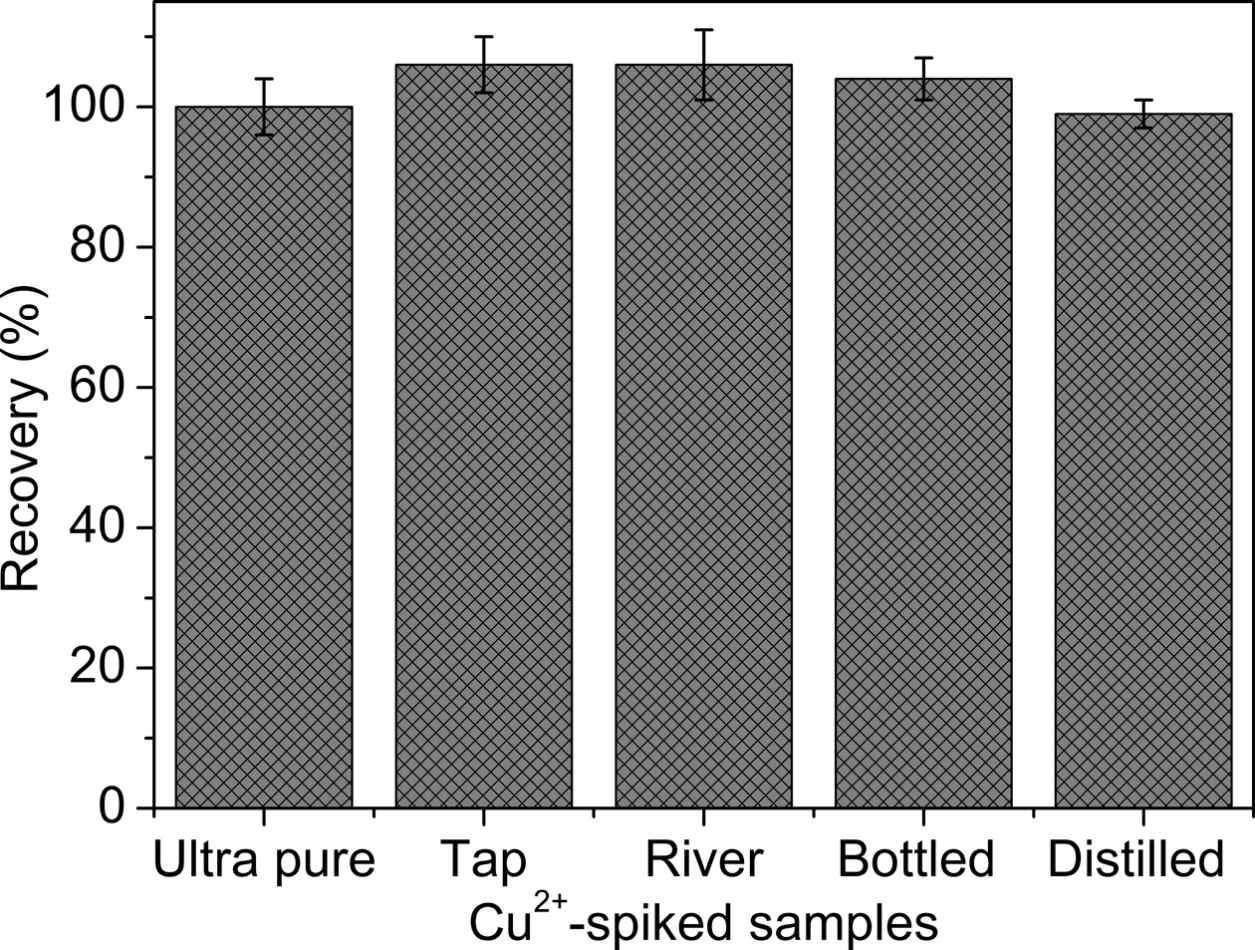
The recoveries of the known amount of Cu^2+^ in Cu^2+^-spiked water samples demonstrates reliability of the established sensing probe.

Cu^2+^ spiked water samples were readily used to determine the copper content and the recoveries of the known amount of Cu^2+^ in the samples were in between 98–110% (Figure 7). The Cu^2+^-spiked water samples (0.96 µM) caused large red-shift in the absorption spectra and dramatic change in visual appearance of yellow to red suggests possibilities in implementing both colorimetric and spectrophotometric measurements (Figure 7). Levels of Cu^2+^ in Cu^2+^-spiked tap water (2.9 μM) and pond water (3.2 μM) were measured using the designed method and compared with literature values obtained by alternative techniques [31]. The tested Cu^2+^ contents were in a reasonable range relative to the literature data discussed in Table 1 for gold NPs [32], and also with atomic absorption spectroscopy [33], and inductively coupled plasma mass spectroscopy [34].

## Conclusion

Functionalized AgNPs were synthesized using casein peptides as the reducing and stabilizing agent in an aqueous medium. The formation of AgNPs and the effect of centrifugation on their stability were verified by measuring the SPR spectra using a UV–vis spectrophotometer. This paper designates the sensing application of water-dispersible casein peptide-capped AgNPs as a sensing probe for the colorimetric detection of Cu^2+^ ions in water samples, and the color change is visible with the naked eye. When compared with alternative reported methods, the proposed procedure is intrinsically more sensitive, due to the direct coordination of divalent Cu ions using the peptide coordination complex. Therefore, the AgNPs–casein peptide system offers a new possibility for the rapid and sensitive real-time detection of Cu^2+^. The AgNPs–casein peptide system holds great potential for developing practical applications to detect Cu^2+^ in various types of drinking and non-drinking water samples, without involving expensive analytical instruments.

## Experimental

### Chemicals

AgNO_3_ and casein peptide (vitamin-free) were purchased from Sigma-Aldrich (Korea). NaOH and NaCl were obtained from Dae Jung Chemicals Korea. Standard solutions, including Cu^2+^, Mn^2+^, Mo^3+^, Na^+^, Cr^3+^, Hg^2+^, Ni^2+^, Ca^2+^, K^+^, Cs^+^, Li^+^, As^+^, PO_4_^3–^, NH_4_, and NO_3_^−^ were obtained from Kanto Chemicals. All reagents were of analytical grade and used without any further treatment. Ultrapure distilled water was prepared freshly and used throughout the synthesis and sensing experiments.

### Synthesis of casein peptide-capped AgNPs

The casein peptide-capped AgNPs were prepared as follows: Briefly, 16.9 mL ultrapure water was added into a series of 50 mL glass tubes, followed by 2 mL casein peptide stock solution (0.6% w/v). This reaction mixture was then preheated at 95 °C before 1 mL AgNO_3_ (20 mM) was added. The freshly prepared AgNPs were purified from the reducing and stabilizing agents (casein peptides) involved in the synthesis, by using centrifugation (10000 rpm for 12 min). The stability of the casein peptide-capped AgNPs was revealed after removal of excess casein peptides from the AgNPs solution, by observing the surface plasmon resonance (λ_max_) and bandwidth (Δλ), before and after centrifugation of the AgNPs.

### Characterization of AgNPs

The UV–vis spectra of the AgNPs in the presence and absence of Cu^2+^ were observed, using an Optizen 2120 spectrophotometer (Korea). To reveal the stability of the centrifuged AgNPs, λ_max_ and Δλ were recorded after each addition of 0.25 mL distilled water to 1 mL of AgNPs stock solution. HR-TEM samples were prepared using carbon-coated copper grids. Shape, size-distribution, and images were observed on Tecnai G^2^ (FEI Company, USA). The XRD measurements were performed using a thin film sample containing the AgNPs (Rigaku Ultima-IV diffractometer, Cu Kα radiation). The centrifuged AgNPs were examined by FTIR spectroscopy using a Thermo Scientific Nicolet iS5 FTIR spectrometer equipped with a attenuated total reflectance tool, in the 1000–4000 cm^−1^ wavelength range at 0.48 cm^−1^ resolution.

### Selectivity

To assess the selectivity of the proposed method, coordination of the functionalized AgNPs was tested with various ions, including Cu^2+^, Mn^2+^, Mo^3+^, Na^+^, Cr^3+^, Hg^2+^, Ni^2+^, Ca^2+^, K^+^, Cs^+^, Li^+^, As^+^, PO^3–^, NH_4_, and NO_3_^−^. Typically, one species was added to distilled water in the presence of 200 μL of AgNPs stock solution at a final concentration of 100 ppb for a 20 min reaction time at ambient temperature, and then, UV–vis spectra were collected in the range 350–800 nm.

### Effect of acidic and alkaline conditions and ionic strength

The effect of acidic and alkaline conditions and ionic strength was studied as follows: 200 μL of the AgNPs stock solution was exposed to different concentrations of diluted HCl (0.1–0.6 mM), NaOH (0.1–0.6 mM), and NaCl (1.0–3.5 mM). The absorbance of the solutions was measured at 410 nm after 5 min. Then, Cu^2+^ (1.28 µM) was added, respectively. The solutions were again incubated for another 5 min before the absorbance of the suspensions was re-measured at 410 nm.

### Detection of Cu^2+^ concentration

The casein peptide-capped AgNPs were used in the sensing experiment at low concentration/optical density (ca. 0.2–0.8 OD). Several different Cu^2+^ concentrations were tested using AgNPs solutions in disposable UV–vis cuvettes after adjusting the volume (1 mL) with distilled water. The total incubation volume of the stock AgNPs solution comprised 0.05, 0.1, and 0.2 mL, respectively, to enable detection over a wide range (0.08–1.44 µM Cu^2+^). After the coordination reaction was performed at 24 °C for 20 min, the spectral shift was detected by UV–vis spectroscopy, and the solution color was observed with the naked eye.

### Real-time UV–vis response of AgNPs toward Cu^2+^

The AgNPs solution (200 µL) was incubated with 0.64, 0.96 and 1.28 µM Cu^2+^ in distilled water. At different reaction times, the stability behavior of the coordination product was studied by measuring the absorption spectra of the reaction solutions. Similarly, the rapid absorbance response of AgNPs aggregates was observed immediately after the Cu^2+^ coordination reaction, and the absorbance of the reaction solutions was measured at 520 nm. Subsequently, the rapid absorbance responses were determined every 5 s for a total time of 30 s at the three different Cu^2+^ concentrations.

### Spiked water samples analysis

UV–vis absorbance of the AgNPs was recorded at 520 nm after reacting various water samples containing Cu^2+^. Typically, 200 μL of the AgNPs solution was treated with Cu^2+^ spiked water samples for 20 min. The absorbance of the AgNP suspensions was recorded after 20 min at 520 nm. A plot of the concentration versus absorbance intensity was prepared, and used as the standard graph for the determination of Cu^2+^ concentrations present in the water samples.
